# Antimicrobial and anti-biofilm activity of essential oils extracted from *Clausena lansium* (Lour.) Skeels and their main constituents against *Streptococcus mutans*

**DOI:** 10.3389/fmicb.2025.1612681

**Published:** 2025-08-29

**Authors:** Zibei Wu, Xufeng Zhang, Lili Wang, Jinlei Sui, Mingqi Yu, Bingzhangke Bao, Jianing Qian, Guohui Yi, Yinzheng Ma, Xiaowen He

**Affiliations:** ^1^School of Pharmacy, Hainan Medical University, Haikou, China; ^2^Department of Stomatology, The First Affiliated Hospital of Hainan Medical University, Haikou, China; ^3^Research Institute for Science and Technology Development, Hainan Medical University (Hainan Academy of Medical Sciences), Haikou, China; ^4^Hainan Provincial Key Laboratory for Tropical Cardiovascular Diseases Research and Key Laboratory of Emergency and Trauma of Ministry of Education, Institute of Cardiovascular Research of the First Affiliated Hospital, Hainan Medical University, Haikou, Hainan, China

**Keywords:** *Streptococcus mutans*, *Clausena lansium*, essential oil, biofilm, dental caries, virulence gene

## Abstract

*Streptococcus mutans* is one of the most significant pathogens leading to dental caries by producing highly cariogenic biofilms within the oral cavity. However, the increasing antimicrobial resistance brings great difficulties to the prevention and treatment of dental caries. Therefore, it is very important to develop new and effective antimicrobial agents from natural sources. *Clausena lansium* (Lour.) Skeels, a plant commonly cultivated in Southern China, is rich in various active constituents. In this study, the main constituents of the essential oils extracted from *Clausena lansium* leaves (EOL), seeds (EOS), peels (EOP) were precisely analyzed by gas chromatography–mass spectrometry. The antimicrobial and anti-biofilm activities of EOL, EOS, and EOP, along with their main chemical constituents, against *Streptococcus mutans* were comprehensively evaluated. The primary constituent in EOL was β-caryophyllene, with a content of 112.29 mg/mL. EOS was found to be abundant in sabinene and 4-terpineol, with constituents of 147.21 mg/mL and 54.36 mg/mL, respectively. In EOP, the most predominant constituent was β-phellandrene, with a content of 173.95 mg/mL. The minimum inhibitory concentrations of EOL, EOS, and EOP were 0.12, 2.00, and 1.00 mg/mL, respectively. The minimum concentrations of inhibiting at least 90% biofilm formation of EOL, EOS, and EOP was 0.12 mg/mL, 2.00, and 2.00 mg/mL, respectively. Intriguingly, the antimicrobial and anti-biofilm efficacies of the main constituents were significantly lower than those of the essential oils. By observing the dynamic changes of the biofilm formation of *S. mutans* treated with essential oils, it was found that the process of biofilm formation was greatly delayed. In some cases, there was even no biofilm formation. The results of real-time quantitative PCR indicated that the expression levels of *brpA*, *gtfB*, *gtfC*, *gtfD*, and *luxS* genes were down-regulated after treatment with the essential oils. This study reinforces the potential of *Clausena lansium* as a promising source for developing new antimicrobial and anti-biofilm agents. They could inhibit the proliferation and virulence of *S. mutans*, thereby contributing to the control of dental caries.

## 1 Introduction

The oral micro-environment maintains a dynamic balance under normal physiological conditions. However, once this equilibrium is disrupted, certain commensal microorganisms have the potential to transform into pathogens, thereby triggering relevant oral disease (Aspinall et al., 2021). Dental caries, a multifactorial oral disease, is one of the most chronic non-communicable diseases afflicting humanity (Aas et al., 2008). The high incidence and extensive prevalence of dental caries cannot be ignored. *Streptococcus mutans* (*S. mutans*), recognized as the most significant pathogens responsible for dental caries, commonly adheres to the tooth surface and gives rise a biofilm (Krzysciak et al., 2014). The dental biofilm, in fact, acts as the primary instigator for the onset of dental caries. Specifically, the biofilm has the ability to bind to the receptors on the bacterial surface. This binding not only enhances the resistance to drugs permeation but also fortifies the adhesion of pathogen to the tooth enamel (Gao et al., 2024).

The adhesion and biofilm formation of *S. mutans* are regulated by several genes, playing a distinct role throughout the biofilm formation process. Glucosyltransferases (Gtfs) and glucan-binding proteins (Gbps) are prominent surface protein antigens that are crucial for the adhesion of *S. mutans* (Zhang et al., 2021; Nakano et al., 2008). There are three Gtfs enzymes, namely GtfB, GtfC, and GtfD, which are, respectively, encoded by the *gtfB*, *gtfC*, and *gtfD* genes. Among four types of Gbps, GbpD encoded by *gbpD* gene is considered as one of the most virulence factors for dental caries. The *luxS* gene governs the synthesis of extracellular glucan, which in turn affects the adhesion ability of *S. mutans* to dental surfaces (Yoshida et al., 2005). The *brpA* gene, which codes for a predicted surface-associated protein, is mainly involved in cell accumulation and biofilm development (Wen et al., 2006). Given these genetic mechanisms, inhibiting biofilm formation is of great importance for the prevention and treatment of dental caries.

Antimicrobial drugs, such as fluoride and chlorhexidine, have been employed to control cariogenic biofilms in the oral cavity. On one hand, these drugs are also associated with several undesirable side effects, including toxicity, tooth discoloration, mucosal irritation, and alterations in taste sensations (Lessa et al., 2010; Walsh et al., 2015; Karpiński and Szkaradkiewicz, 2015). On the other hand, the prolonged administration of antibiotics can disrupt the balance of the oral flora and lead to the development of antimicrobial resistance (Liao et al., 2015; Cieplik et al., 2019). Consequently, current studies on the prevention and treatment of dental caries has shifted its focus toward the discovery of natural antimicrobial and anti-biofilm agents. These agents are expected to be effective, safe, and suitable for long-term use (Pallavi et al., 2024; Madiba et al., 2023; Liu et al., 2023).

*Clausena lansium* (Lour.) Skeels is a small evergreen tree belonging to the Rutaceae family. It is indigenous to southern China and widely cultivated in regions such as Guangdong, Guangxi, Hainan, Taiwan, and Fujian. Currently, it has been introduced to tropical and subtropical areas across the globe. In China, Vietnam, and Thailand, the fruits of *Clausena lansium* can be eaten fresh or processed into jams, beverages, pies, and dried snacks (Lim, 2012). The leaves, seeds, and peels of *Clausena lansium* have long been utilized as herb medicines to address a variety of ailments, including sore throat, rheumatism, joint pain, malaria, scabies, and snake bites (Li et al., 1991; Shen et al., 2017; Yan et al., 2018; Fan et al., 2018). It exhibits remarkable antimicrobial, antifungal, antimalarial, and anti-inflammatory properties. As reported in the literature, *Clausena lansium* is rich in a diverse range of active components, such as essential oils, alkaloid, coumarins, and flavonoids (Adebajo et al., 2009; Shen et al., 2012). In our previous study, essential oils extracted from leaves (EOL), seeds (EOS), peels (EOP) of *Clausena lansium* by hydrodistillation were analyzed via gas chromatography–mass spectrometry (GC–MS) using the peak areas without applying correction factors. All the essential oils (EOs) had anti-*Candida* activity by the disk diffusion method, and EOS showed the most potent effect (He et al., 2019; Ma et al., 2021). Subsequently, a further study of quantitative analysis of five volatile constituents in EOS was performed by GC–MS using a standard curve method (Ma et al., 2023). However, the main volatile constituents in EOL and EOP of *Clausena lansium*, as well as anti-*S. mutans* activity of EOL, EOS, and EOP, remain to be investigated.

In this study, a precise quantitative analysis was conducted on the five main chemical constituents in EOL, EOS and EOP. The antimicrobial and anti-biofilm activities of EOL, EOS, EOP and their main constituents against *S. mutans* were evaluated using the microdilution method and XTT method. Furthermore, the relationship between the main chemical constituents and antimicrobial activity of EOL, EOS, and EOP was also evaluated. The expression level of virulence genes related to biofilm formation of *S. mutans* treated with EOL, EOS, and EOP at the concentration of MIC and 1/2 MIC were detected by real-time quantitative PCR (RT-qPCR). These genes included *brpA*, *gbpD*, *gtfB*, *gtfC*, *gtfD*, and *luxS*. The objective of the study is to reveal the mechanism of anti-biofilm activity of essential oils extracted from *Clausena lansium* against *S. mutans* on the changes of virulence gene expression and to provide a basis for the development of these EOs as natural agents for combating dental caries.

## 2 Materials and methods

### 2.1 Bacterial strains and cultivation

*Streptococcus mutans* UA159 was purchased from Shanghai Ruichu Biotech Co., Ltd. (Shanghai, China). It was first inoculated onto Brain Heart Infusion Agar (BHIA) (Guangdong Huankai biotech Co, Ltd., Guangdong, China) and incubated in the CO_2_ incubator (Shanghai Boxun Industrial Co., Ltd., Shanghai, China) at 37°C with a 5% CO_2_ atmosphere for 24–48 h. Subsequently, a single colony was inoculated into 10 mL Brain and Heart Infusion (BHI) broth (Guangdong Huankai biotech Co, Ltd., Guangdong, China), which was then incubated at 37°C until logarithmic growth phase.

### 2.2 Plant materials and chemicals

*Clausena lansium* (Lour.) Skeels leaves, seeds, and peels were collected from Tunchang, Hainan, China, in June 2022 and identified by Professor Weili Yang (School of Pharmacy, Hainan Medical University) and stored in Public Research Center, Hainan Medical University (Haikou, China). β-Caryophyllene (>90%) was supplied from TCI Chemical Industry Development Co., Ltd. (Shanghai, China). Sabinene (>75%), 4-terpineol (>98%) and *α*-phellandrene (>85%) were supplied by Sigma-Aldrich (Shanghai, China). β-Phellandrene (>96%) was purchased from Toronto Research Chemicals TRC (Toronto, Canada).

### 2.3 Extraction and analysis of EOs

*Clausena lansium* leaves were dried in shade. After removing the pulps of fruits, seeds and pericarps were washed and then dried in an oven at 40–50°C. These leaves, seeds and pericarps were roughly crushed, and soaked in water with a material-to-solvent ratio of 1:10 (*w/v*) for 5.0 h. EOL, EOS, and EOP were extracted by hydrodistillation for 2.5–3.0 h in a Clevenger-type apparatus. The obtained EOs were stored at 4°C in an air-tight container and dried using anhydrous sodium sulfate before being analyzed. According to our pervious study (Ma et al., 2023), five chemical constituents in these EOs, including sabinene, *α*-phellandrene, β-phellandrene, 4-terpineol, and β-caryophyllene, were analyzed by GCMS-QP2010 Plus (Shimadzu, Kyoto, Japan) using a standard curve method.

### 2.4 Antimicrobial activity

The antimicrobial activity of EOL, EOS, EOP and the four main chemical constituents (β-caryophyllene, sabinene, 4-terpineol and β-phellandrene) against *S. mutans* was determined using the microdilution method according to guideline of M100-S24 (Clinical and Laboratory Standards Institute, 2014). EOs and chemicals were initially reconstituted in dimethyl sulfoxide (DMSO) (Thermo Scientific Co., Ltd., Shanghai, China) and diluted with BHI broth to a final concentration in the range of 16–0.03 mg/mL with DMSO <1%. A solution containing 0.1% DMSO was prepared as a negative control. Chlorhexidine acetate (CHX) (Aladdin, Shanghai, China) was used as the positive control. The inoculum of *S. mutans* was adjusted to a concentration of 1.5 × 10^6^ CFU/mL. Except for the blank control group, to which no bacterial suspension was added, the bacterial suspension was added to all other samples. The prepared 96-microwell plate was incubated at 37°C with a 5% CO_2_ atmosphere for 16–24 h. Finally, after observing, the wells with no visible colonies and remaining a clear state were identified as the minimum inhibitory concentrations (MICs). To determine the minimum bactericidal concentrations (MBCs), 100 μL of the culture from the clear and transparent wells, as well as the blank control wells, was transferred onto the BHIA plates and further incubated at 37°C for 24 h. All the operations were performed in triplicate.

### 2.5 Anti-biofilm activity

The anti-biofilm activity of EOL, EOS, EOP and the four main chemical constituents against *S. mutans* was determined using 2,3-bis(2-methoxy-4-nitro-5-sulfophenyl)-5-[(phenylamino)carbonyl]-2H-tetrazolium hydroxide (XTT) method. The solutions of EOs and chemicals were prepared with five concentrations (1/4MIC, 1/2MIC, MIC, 2MIC, 4MIC). A solution containing 0.1% DMSO was prepared as a negative control. Except for the blank control group, to which no bacterial suspension was added, the bacterial suspension was added to all other samples. The prepared 96-microwell plate was incubated with a 5% CO_2_ atmosphere at 37°C for 48 h to form biofilm. Then, each well was rinsed twice to three times using the sterile PBS (0.01 mol/L, pH7.4) (Shanghai Yuanye Biotech Co., Ltd., Shanghai, China). 100 μL of mixed solution with XTT sodium salt and phenazine methosulfate (PMS) (Aladdin, Shanghai, China) was added. After incubation in dark at 37°C for 4 h, the OD values at 490 nm were measure by Synergy HTX microplate reader (BioTek, USA). All the operations were performed in triplicate. The inhibition rate is calculated according to the following formula:

Inhibition rate (%) = [OD_490_ (negative control) − OD_490_ (sample)] / [OD_490_ (control) − OD_490_ (blank control)] × 100

The minimum concentration to inhibit at least 90% biofilm formation (MBIC_90_) of EOs and the main constituents against *S. mutans* was also calculated.

### 2.6 Observation of biofilm formation

To evaluate the impact of EOL, EOS, and EOP on the biofilm formation of *S. mutans*, Lionheart FX Automated Live Cell Imager (BioTek, USA) was employed to conduct the real-time monitoring the process of biofilm formation. A 96-microwell plate was prepared with the tested EOs at the concentrations of 1/2MIC, MIC and MBC. A BHI culture medium containing 0.1% DMSO was used as an untreated control. Except for the blank control group, to which no bacterial suspension was added, the bacterial suspension was added to all other samples. The plate was incubated with a 5% CO_2_ atmosphere at 37°C for 48 h. The whole process of biofilm formation was observed and recorded at a magnification of 4 × every 30 min. Three duplicate wells for each group were observed.

### 2.7 Determination of the expression level of virulence genes

The gene expression experiments were carried out to determine whether the EOs could alter the expression of candidate virulence genes associated with biofilm formation of *S. mutans*. *S. mutans* were treated with three EOs at the concentrations of 1/2MIC, MIC. A solution containing 0.1% DMSO was prepared as an untreated control. After inoculation in the CO_2_ incubator at 37°C for 0.5 h, the bacterial suspension was centrifuged at 8000 rpm and 4°C for 1 min using Centrifuge 5424R (Eppendrof, Germany).

The obtained bacterial pellets were rinsed twice with DEPC-treated ddH_2_O (Shanghai Sangon Biotech Co., Ltd., Shanghai, China). Bacterial RNA was extracted following the instruction of the Total RNA Extraction Kit (Shanghai Sangon Biotech Co., Ltd., Shanghai, China). Lysoenzymes were used to react with Buffer Rlysis-B employed to expedite the bacterial lysis process. Subsequently, chloroform extraction and anhydrous ethanol precipitation methods were employed for a more refined RNA extraction. The concentration and purity of the total RNA were assessed by measuring the absorbance ratio at OD_260/280_ using a Synergy HTX microplate reader.

Reverse transcription was used to generate complementary DNA (cDNA), enable amplification and quantification by quantitative polymerase chain reaction (qPCR) method. The experiment was carried out step by step according to the description of the MightySccript first-strand cDNA synthesis Master Mix Kit (Shanghai Sangon Biotech Co., Ltd., Shanghai, China) on Biometra TADVANCED 96SG (Analytik Jena, Germany).

The expression level of virulence genes related to biofilm formation of *S. mutans* treated with EOs of MIC and 1/2 MIC were detected by RT-qPCR, such as *brpA*, *gbpD*, *gtfB*, *gtfC*, *gtfD*, and *luxS*. The primers of RT-qPCR provided by Shanghai Sangon Biotech Co., Ltd. (Shanghai, China) were shown in Table 1, in which *16S rRNA* was the internal reference. Finally, the RT-qPCR reaction was performed according to the description of the SGExcel UltraSYBR Master Premix Solution (Shanghai Sangon Biotech Co., Ltd., Shanghai, China) on Agilent Stratagene Mx3005P (Agilent, USA).

**Table 1:** Primer sequences used in *S. mutans* RT-qPCR.

Gene	Primer (5′-3′)	primer length/bp
*16S rRNA*	F: GTGTGCTCTGGAAACTGTCTG	165
	R: ATCTAATCCTGTTCGCTACCC	165
*brpA*	F: AAGGGCTGGTTCAGTTGG	217
	R: ATGACCTCACGTTGACGCT	217
*gbpD*	F: GATGTGGACCAATCGCTTT	213
	R: GTTCGGCTTGTCTTTCTCAGT	213
*gtfB*	F: CTACACTTTCGGGTGGCTT	134
	R: TTCTTGCTTAGATGTCGCTTC	134
*gtfC*	F: GCGGTTACAGAATCTCAGGC	116
	R: ACAGTGGCGGTTGGTTGA	116
*gtfD*	F: CTGATTCGTGGTATCGTCCTA	194
	R: ACTTTCTTGGCTGCTGTCTG	194
*luxS*	F: AAGCCCCTTATGTCCGTCT	179
	R: ACAGTCAATCATCCCGTCAA	179

### 2.8 Statistical analysis

All experiments were conducted in triplicate, and the results were presented as the mean ± standard deviation. Statistical analysis and data visualization were performed using Prim 6.0 software (GraphPad Software Inc., CA, USA). The comparing test between 2 groups were analyzed by two-way analysis of variance (ANOVA). In the RT-qPCR of *S. mutans*, the relative gene expression of each target gene was quantified using the 2^-ΔΔct^ method.

## 3 Results

### 3.1 Extraction yields and analysis of the main chemical constituents

**Figure 1:**
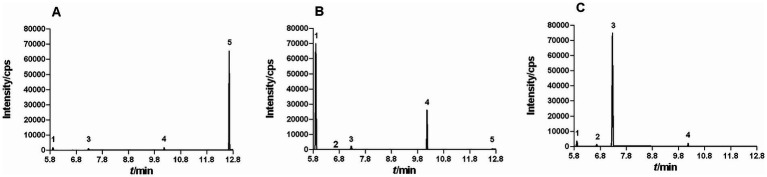
GC–MS Chromatograms of EOL **(A)**, EOS **(B)**, and EOP **(C)** by GC–MS. (1, sabinene; 2, *α*-phellandrene; 3, β-phellandrene; 4, 4-terpineol; 5, β-caryophyllene).

EOL, EOS, and EOP were extracted by hydrodistillation, with the yields of 0.46 ± 0.03%, 0.65 ± 0.03%, and 1.32 ± 0.08% (*v/w*), respectively. These EOs differed in color. EOL presented a yellow hue, EOS was faint yellow, and EOP was colorless. Moreover, the contents of the five constituents in these EOs were also varied as analyzed by GC–MS. The chromatograms of EOL, EOS, and EOP was depicted in Figure 1.

From Table 2, the primary constituent in EOL was β-caryophyllene, with a content of 112.29 mg/mL. EOS was found to be abundant in sabinene and 4-terpineol, with constituents of 147.21 mg/mL and 54.36 mg/mL, respectively. In EOP, the most predominant constituent was β-phellandrene, with a content of 173.95 mg/mL. The main component in the three EOs was quite different. Since the content of *α*-phellandrene in these EOs was extremely low, only the antimicrobial activities of the four main constituents were tested in the following experiment.

**Table 2:** Results of the contents of the five constituents in EOL, EOS, and EOP.

constituent	EOL	EOS	EOP
sabinene	0.27 ± 0.03	147.21 ± 3.21	1.95 ± 0.08
α-phellandrene	-	0.97 ± 0.04	2.18 ± 0.12
β-phellandrene	1.27 ± 0.05	6.77 ± 0.35	173.95 ± 4.06
4-terpineol	1.55 ± 0.06	54.36 ± 2.14	1.82 ± 0.07
β-caryophyllene	112.19 ± 3.47	0.13 ± 0.01	-

“-”: no detected.

### 3.2 Antimicrobial efficacy

The MICs and MBCs of EOs, the main chemical constituents, and CHX against *S. mutans* is presented in Table 3. The MIC values of EOL, EOS, EOP and CHX were 0.12, 2.00, 1.00 and 0.25 mg/mL, respectively. The MBC values were twofold of the corresponding MICs. The MIC values of β-caryophyllene, sabinene, 4-terpineol, and β-phellandrene were all 2.00 mg/mL and the MBC values were much higher than MICs. The MICs and MBCs of EOs were lower than the main chemical constituents, which indicated that EOs had better antimicrobial activities.

**Table 3:** Antimicrobial efficacy of EOL, EOS, EOP and their main chemical constituents against *S. mutans.*

EOs and chemical constituents	MIC/(mg/mL)	MBC/(mg/mL)
EOL	0.12	0.25
EOS	2.00	4.00
EOP	1.00	2.00
β-caryophyllene	2.00	>16.00
Sabinene	2.00	8.00
4-terpineol	2.00	8.00
β-phellandrene	2.00	8.00
CHX	0.25	0.50

### 3.3 Anti-biofilm efficacy

Table 4 presents the inhibitory rates of EOs and their main chemical constituents on the biofilm formation of *S. mutans*. Notably, EOL demonstrated the most remarkable inhibitory activity on biofilm formation of *S. mutans*. It had an MBIC_90_ value of 0.12 mg/mL (equivalent to the MIC), accompanied by an inhibitory rate of 95.23%. The MBIC_90_ values of EOS and EOP were both 2.00 mg/mL, with corresponding inhibitory rates of 96.67 and 99.21%, respectively. For 4-terpineol, sabinene and β-phellandrene, relatively high MBIC_90_ values of 8.00 mg/mL were observed. Their inhibitory rates of 97.53, 91.99 and 91.01%, respectively. The inhibitory rate of β-caryophyllene at a concentration of 8.00 mg/mL was 50.53%. Considering their potentially high MBIC_90_ values against *S. mutans*, these values were not determined in this study.

**Table 4:** The inhibitory rates of EOL, EOS, EOP and their main chemical constituents on the biofilm formation of *S. mutans* (%).

EOs and chemical constituents	1/4MIC	1/2MIC	MIC	2MIC	4MIC
EOL	64.02 ± 0.80	85.25 ± 0.62	95.23 ± 0.73	100.65 ± 0.28	100.02 ± 0.92
EOS	37.98 ± 0.45	46.48 ± 0.71	96.67 ± 0.40	100.10 ± 0.56	100.23 ± 0.90
EOP	63.74 ± 0.66	69.04 ± 0.39	88.72 ± 0.28	99.21 ± 0.46	99.35 ± 0.56
β-caryophyllene	12.48 ± 0.87	20.33 ± 0.47	32.96 ± 0.45	37.27 ± 0.75	50.53 ± 1.34
Sabinene	4.60 ± 0.66	7.34 ± 0.94	28.24 ± 0.51	40.76 ± 1.11	91.99 ± 0.77
4-terpineol	5.26 ± 0.76	13.95 ± 0.76	29.73 ± 1.11	52.09 ± 0.45	97.53 ± 0.86
β-phellandrene	1.48 ± 0.40	5.02 ± 0.59	25.03 ± 0.49	43.04 ± 1.00	91.01 ± 0.89

### 3.4 Effects of EOL, EOS, and EOP on biofilm formation of *Streptococcus mutans*

**Figure 2:**
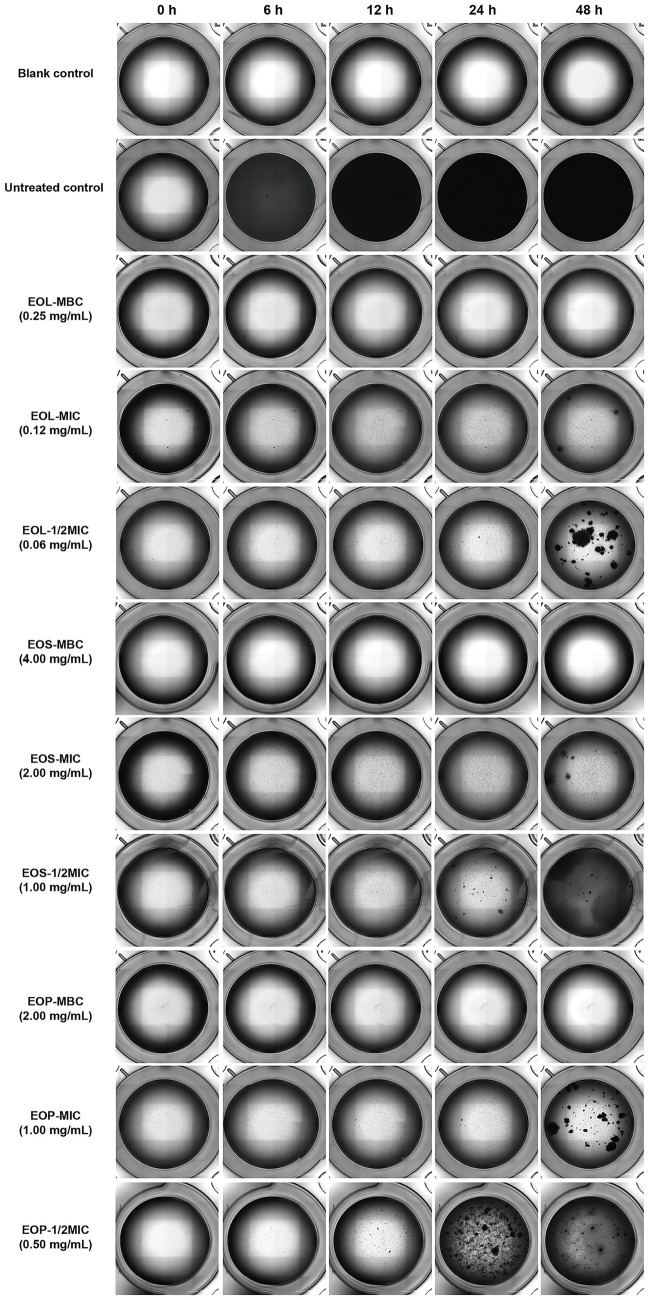
Effects of EOL, EOS, and EOP on biofilm formation of *S. mutans.*

The biofilm formation of *S. mutans* under the concentrations of 1/2MIC, MIC, and MBC of EOL, EOS, and EOP was monitored through photograph every 30 min. The dynamic videos documenting the biofilm formation process of *S. mutans* treated with EOL, EOS, and EOP are provided in the attachment (Appendix A). Representative images captured at 0, 6, 12, 24, and 48 h are presented in Figure 2. In the untreated control group, *S. mutans* grew rapidly and had covered the bottom of the wells at 6 h. In contrast, when *S. mutans* was exposed to EOL, EOS, and EOP at various concentrations, these essential oils demonstrated the potential to impede the biofilm formation process and suppress its initiation. Moreover, there was a clear dose-dependent relationship between the concentration of the EOs and their inhibitory effect. As the concentrations of the EOs increased, their ability to inhibit biofilm formation became more pronounced. When the EOs were applied at their MBCs, the growth of *S. mutans* was undetectable.

### 3.5 Effects of EOL, EOS, and EOP on the expression of virulence genes

**Figure 3:**
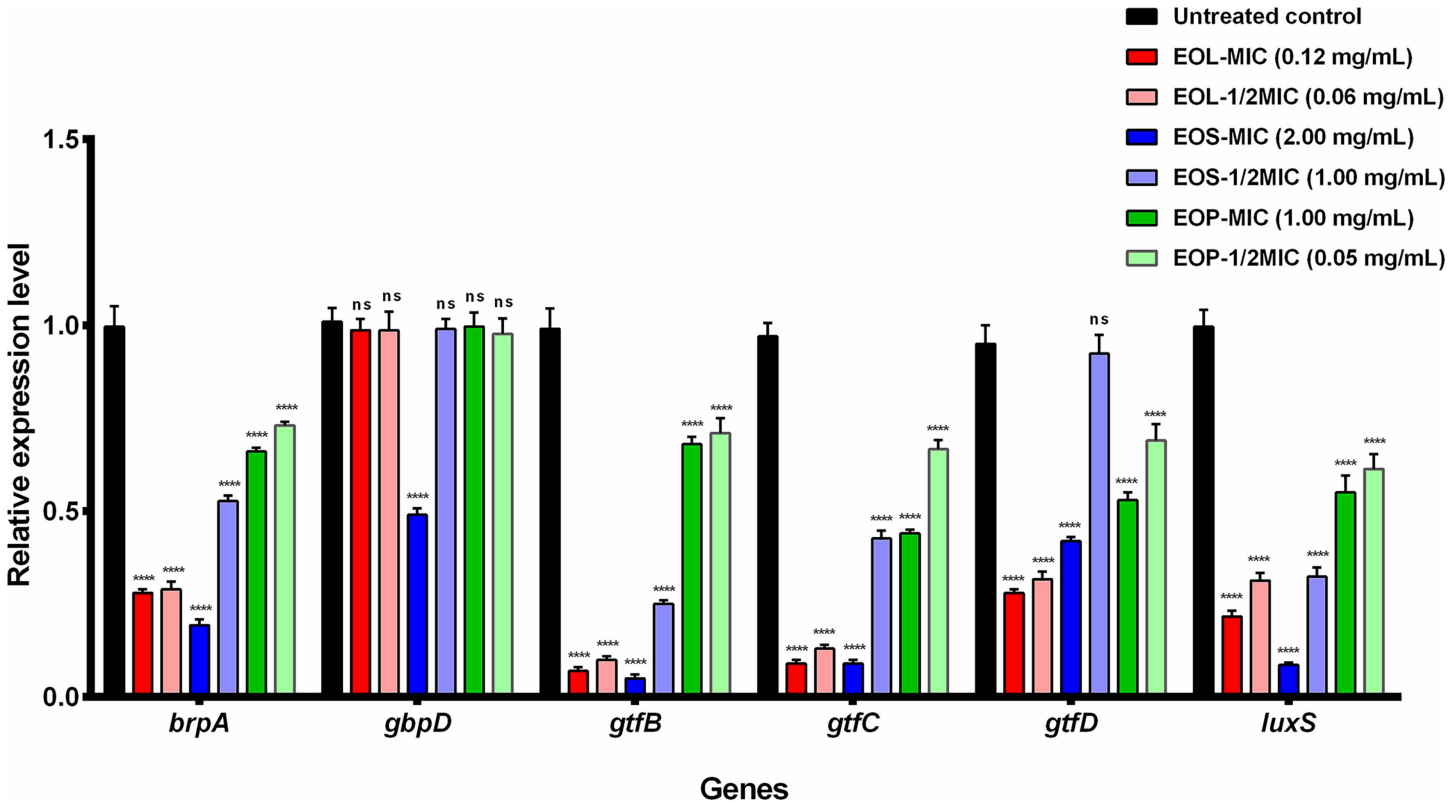
The relative expression of genes treated with EOL, EOS, and EOP (^ns^ *p* ˃ 0.05, ****p < 0.0001 *vs* the control).

Total RNA was extracted, and the values of OD_260/280_ were ranged from 1.80 to 2.20, suggesting that the RNA quality was stable and met the requirements for reverse transcription. The relative expression levels of the virulence genes were calculated and are depicted in Figure 3. When compared to the control group, a significant decrease in the expression of most target genes was observed after treatment with EOs. Only for gene *gbpD*, there was no significant difference (*p* ˃ 0.05) except when treated with MIC of EOS, which led to a significant downregulation (*p* < 0.0001). For the gene *gtfD*, no significant difference was detected in the treatment with 1/2MIC of EOS (*p* ˃ 0.05). Furthermore, as the concentration of EOs increased, the degree of gene downregulation became more prominent.

## 4 Discussion

Dental caries has posed substantial threats to oral health. Currently, various antimicrobial drugs such as fluoride and chlorhexidine are available on the medical market for the prevention and treatment of dental caries. However, the emergence of fluorine-resistant oral bacteria, along with the side-effects and cytotoxicity of chlorhexidine, has restricted the application scope of these drugs (Liao et al., 2015; Karpiński and Szkaradkiewicz, 2015; Cieplik et al., 2019). In contrast, research on drugs extracted from natural plants has attracted increasing attention, because they are favored for their potent antimicrobial characteristics and relatively fewer adverse effects (Pallavi et al., 2024; Madiba et al., 2023; Liu et al., 2023).

*Clausena lansium*, a plant resource with both as medicinal and edible values, possesses diverse pharmacological functions in traditional Chinese medicine. It can be used for treating various ailments, including sore throat, rheumatism, joint pain, scabies, snake bites. In this study, we mainly focus on the quantitative analysis of the EOs and their antimicrobial and anti-biofilm effects against *S. mutans.* The EOs including EOL, EOS, and EOP were extracted and the colures were varied. This indicated that the constituents of EOs may be also different. Hence, a further study of quantitative analysis of five main constituents in EOs was performed by GC–MS. The results showed that the primary constituent in EOL was β-caryophyllene. EOS was found to be abundant in sabinene and 4-terpineol. In EOP, the most predominant constituent was *β*-phellandrene. Obviously, there were obvious differences in the main constituents of the EOs. Thereafter, the antimicrobial activities of the three EOs and the four primary components were assessed using the microdilution method. Nevertheless, it was found that the antimicrobial efficacy of all the main chemical constituents was lower than that of the EOs. Among all the EOs, the EOL exhibited the most potent antimicrobial activity, outperforming the positive control, CHX.

A previous research has indicated that β-caryophyllene had anti-biofilm activity against *S. mutans* by reducing the expression of *gtf* genes (Yoo and Jwa, 2018). Moreover, it had antimicrobial efficacy against periodontopathogens, including *Porphyromonas gingivalis, Tannerella forsythia,* and *Treponema denticola* (Yoo and Jwa, 2019). Additionally, sabinene had been proven to effectively suppress the growth and adherence of *S. mutans* and impede the development of its biofilm by inhibiting cariogenic virulence factors (Park et al., 2019). However, in our study, the anti- *S. mutans* effects of β-caryophyllene and sabinene were not as potent as those of the EOs. Through a comparison of the antimicrobial efficacy between *Clausena lansium* EOs and their main components in this research, it indicated that the inhibitory effects of the EOs against *S. mutans* may not solely rely on their main components. Instead, these effects might be attributed to the complex components of the EOs, or the synergistic antimicrobial properties they exhibited.

*Streptococcus mutans* commonly adheres to the tooth surface and forms a biofilm, which serves as one of the primary factors in the development of dental caries. In light of this, the anti-biofilm activities of all the EOs and their main chemical constituents were determined using XTT method and real-time microscopic observation. The results indicated that all of them can inhibit the formation of biofilm in *S. mutans.* Moreover, the inhibitory rates increased in proportion to the rising concentration of EOs and their main constituents. But the inhibitory effect of each individual constituent on biofilm formation was significantly weaker than that of the EOs. These findings further corroborated the hypothesis that the antimicrobial and anti-biofilm effects of the EOs extracted from *Clausena lansium* may be not solely attributed to their main constituents in EOs, but rather to the synergistic effect of multiple constituents.

To further elucidate the antimicrobial mechanism of EOs against *S. mutans*, the expression of virulence genes was evaluated in the present study. From the perspective of genes, *brpA*, *gtfB*, *gtfC*, *gtfD*, and *luxS* genes are related to the adhesion and biofilm formation. In previous reports, the EOs of *Origanum vulgare* L. and *Origanum heracleoticum* L. significantly repressed the expression of genes, including *gtfB*, *gtfC*, *gtfD*, *spaP* and *gbpB*, which are predominantly involved in extracellular polysaccharide synthesis and biofilm formation, thereby hindering the growth of *S. mutans* (Yuan et al., 2023). Lemon EOs can reduce the adherence of *S. mutans* by suppressing the transcription of *gtfB*, *gtfC*, *gtfD*, thus diminishing the activity of GTFs. Furthermore, this oil can mitigate the acid tolerance and biofilm formation of *S. mutans* by down-regulating the expression of *luxS* and *srtA* genes, which are crucial for biofilm formation and acid resistance (Sun et al., 2017). In this study, the expression level of virulence genes *brpA*, *gbpD*, *gtfB*, *gtfC*, *gtfD*, and *luxS* in *S. mutans* treated with EOs at MIC and 1/2 MIC were detected. The results showed that the inhibitory effect was enhanced as the concentration of the EOs increased. EOL exerted the most significant inhibitory effect on the expression of genes. EOS demonstrated a moderate inhibitory effect, while EOP exhibited the least inhibitory impact. These outcomes were consistent with the findings obtained from the XTT method. The EOs down-regulated the expression of genes *brpA*, *gtfB*, *gtfC*, *gtfD*, and *luxS.* This may reduce the adhesion ability of *S. mutans* and cause defects in biofilm accumulation, thereby influencing biofilm formation.

In conclusion, the present study investigated the specific contents of the five main constituents in the EOs from *Clausena lansium*, as well as the antimicrobial and anti-biofilm activities of the EOs and their main constituents against *S. mutans*. The EOs exhibited better potent antimicrobial and anti-biofilm effects than their main constituents. They can inhibit the growth of *S. mutans* by down-regulating the expression of virulence genes, such as *brpA*, *gtfB*, *gtfC*, *gtfD*, and *luxS*. This study may elucidate the anti-*S. mutans* mechanism of *Clausena lansium* EOs through suppressing the proliferation and virulence of *S. mutans*, and hence preventing dental caries. It provides a theoretical basis for the development of natural antimicrobial and anti-biofilm agents for the control of dental caries.

## Data Availability

The datasets presented in this study can be found in online repositories. The names of the repository/repositories and accession number(s) can be found in the article/Supplementary material.
