# Supplementary material for: Antimicrobial and anti-biofilm activity of essential oils extracted from Clausena lansium (Lour.) Skeels and their main constituents against Streptococcus mutans
Source: Front Microbiol. 2025 Aug 29;16:1612681. doi: 10.3389/fmicb.2025.1612681 (PMC12425991; doi:10.3389/fmicb.2025.1612681)
Supplement: Supplementary file 1 [file Table_1.DOCX]

**Appendix A legends:** Dynamic videos documenting the biofilm formation process of *S. mutans* treated with EOL, EOS, and EOP

Video 1-blank control (only BHI culture)

Video 2-untreated control

Video 3-treated with 0.25 mg/mL of EOL (MBC)

Video 4-treated with 0.12 mg/mL of EOL (MIC)

Video 5-treated with 0.06 mg/mL of EOL (1/2MIC)

Video 6-treated with 4.00 mg/mL of EOS (MBC)

Video 7-treated with 2.00 mg/mL of EOS (MIC)

Video 8-treated with 1.00 mg/mL of EOS (1/2MIC)

Video 9-treated with 2.00 mg/mL of EOP (MBC)

Video 10-treated with 1.00 mg/mL of EOP (MIC)

Video 11-treated with 0.50 mg/mL of EOP (1/2MIC)
